# Back to Locality? Demand Potential Analysis for Short Food Supply Chains

**DOI:** 10.3390/ijerph20043641

**Published:** 2023-02-18

**Authors:** Krzysztof Solarz, Magdalena Raftowicz, Marian Kachniarz, Agnieszka Dradrach

**Affiliations:** 1 Doctoral School, Wroclaw University of Environmental and Life Sciences, 50-357 Wrocław, Poland; 2 Department of Applied Economics, Wroclaw University of Environmental and Life Sciences, 50-363 Wrocław, Poland; 3 Institute of Spatial Management, Wroclaw University of Environmental and Life Sciences, 50-375 Wrocław, Poland; 4 Institute of Agroecology and Plant Production, Wroclaw University of Environmental and Life Sciences, 50-363 Wrocław, Poland

**Keywords:** demand potential analysis, short food supply chains, local food, Poland

## Abstract

The main purpose of the article is an attempt to estimate the demand for products traded within short food supply chains in Poland. The survey was conducted in autumn 2021 in the Kamienna Góra county, where the first business incubator in Poland, addressed to farmers and food producers, initiated and supported by the local government, is located. The Computer-Assisted Web Interview (CAWI) method constituted the basis for the process of collecting research material. The channel for reaching respondents was the LIBRUS application and local social media. Responses were mainly given by women, people with incomes in the range of PLN 1000–3000 per person, those aged 30–50, and those with a university education. The research results showed a high level of potential demand for local agri-food products, which should encourage farmers to switch from long to short supply chain models. The persisting low awareness regarding the existence of alternative distribution networks for local products, which primarily requires increased activities in the field of territorial marketing that promote local agri-food products among the residents of municipalities constitutes, from the perspective of consumers, a barrier to the development of short food supply chains.

## 1. Introduction

The growing demand for local food has resulted in increased interest in alternative agri-food chains. One of the dynamically developing research directions is the analysis of consumers’ motivations, attitudes, and inclinations to purchase local products [1]. The organization of these local markets is carried out within the framework of short food supply chains (SFSCs) perceived as an alternative distribution model, based on three types of “proximity” occurring between trade participants: physical (locality), organizational (reducing the number of links within the chain), and social (communication, trust, knowledge, and flow of information about the product between a producer and a consumer) [2]. In a broad sense, shortening food supply chains is even approached as an element of increasing food security [3,4] and improving the quality of life [5,6].

Poland is characterized by a highly fragmented structure of farms—as many as 72% do not exceed the area of 5 ha. These are predominantly small family farms engaged in small-scale production, based on traditional farming methods. The smallest farms in Poland (up to and including 2 ha of UAA) account for over 1/5 of all farms [7]. Therefore, it seems to be a very convenient market for the development of SFSCs. It turns out, however, that thus far, the interest in such forms of sales has been small. Despite the formal facilitation introduced in 2017, regulating small-scale processing and sales, it is estimated that only 2.11% of farmers in Poland took advantage of them in 2017–2021.

In the face of such disproportions in the number of agricultural producers interested in selling their products between Poland and the other European Union countries, the question arises: is there a demand potential in Poland for the development of short food supply chains? Unfortunately, the existing research does not bring us closer to this answer, because, in Polish scientific literature, the vast majority of studies are focused on the supply potential only [8,9,10,11,12,13]. It seems, therefore, that there is a need to develop demand analyses highlighting the needs reported by the consumers.

The main purpose of the presented research is an attempt to fill the existing gap by estimating the demand for products traded within short food supply chains. The subject of this research refers to the preferences and interests of consumers focused on the local food market. Therefore, the research area was narrowed down to the local scale (single-county area). The choice of the research location was not a random one—it is the area where the first rural business incubator in Poland was established, addressing its activities to farmers and food producers, initiated and supported by the local government.

## 2. Background

The problems of supply chains are discussed by many scientific studies. Most often, supply chains are referred to as systems of business relations referring to the product, through which goods move from the place of production to consumption at a certain time [14,15].

Food supply chains constitute a particular issue in the analysis of supply chains. The food economy is one of the most important and, at the same time, the most complex segments of the global economy system. It results from the fact that food is an essential product in the human consumption process, having no substitutes, which makes its price elasticity rigid in the short term [3,4].

Thus far, a universal and commonly used definition of the food supply chain has not been developed. Noteworthy is the definition of S. Dani, who understands the food supply chain as a “combination of processes, operations, and entities that transform food from its raw state to food on the plate” [16]. This definition emphasizes that the entities cooperate with each other within the food supply chain and create a network of connected elements to provide consumers with a specific product or service.

Demand is a key category in the supply chain concept, while the knowledge and ability to anticipate demand allow the smooth functioning of the whole organization, and consequently, the entire chain. This thesis is confirmed by M. Holweg and F.K. Pil [17], who recognized, taking a subjective approach, i.e., that it is the consumer who is the driving force of the supply chain, instead of an objective approach—the market, or rather the demand for a product that meets the needs of buyers and constitutes the subject of the actual flow in this chain. It follows that the demand should play a decisive role in the structure of the chain, its flexibility as well as the time and form of product delivery. Unfortunately, as rightly noted by N. Szozda and A. Świerczek [18], the awareness of the key role of demand in supply chains, both in theory and practice, remains highly superficial and difficult to implement, as it requires the following activities from the individual entities operating in the supply chain [19]:

- Continuous data collection on consumer tastes and preferences;
- Establishing and strengthening long-term relationships with clients;
- Monitoring and assessment of the level of services provided to clients, as well as the degree of their satisfaction.

Having recognized the priority role of demand, many definitions of the supply chain emphasize the role of the end user (consumer), who is the crucial link in the chain towards which the flow of products and services takes place, from the original source, through all intermediate forms on the way. Following this approach, the primary goal of the supply chain is to meet customer requirements in terms of service quality at the lowest possible cost [20].

Consumer preferences in the short food chain market are relatively rarely analyzed. Most often, these studies cover either regions or countries with an established position in the short supply chain market, such as France or Great Britain. Still, only a few studies have been published regarding the assessment of the demand potential for products purchased in short food supply chains. A specific asymmetry of research can be observed in this respect [21,22], as the available studies usually focus on the analysis of supply [23].

France is the exception. The abundance of statistical data in France results from the fact that the census there includes questions about short food supply chains [24], which is unique on a global scale. In France, between 6 and 7% of food purchases are made within short supply chains [25]. However, the research by D. Gallaud and B. Laperche [26] showed that French farmers are convinced that consumers are still insufficiently motivated to allow producers the full switch into short food supply chains. In turn, consumer research carried out in 2013 showed that as many as 42% of the respondents bought at least one product in short food supply chains in the month preceding the study [24]. The research by A.W. Gilg and M. Battershill [27] conducted in north-western France in 2000 showed that the short supply chain market is focused only on certain niche products that consumers are interested in, and on farmers who want to avoid modern agriculture based on “high technologies”.

On the demand side, it is worth noting that the consumers buying products within short supply chains reevaluate their purchasing attitudes from pro-quantity to pro-quality, which enables them to make new judgments regarding the value of food based on their knowledge, experience, or image. The research by P. Mundler and S. Laughrea showed that sales in short food supply chains allowed reducing the prices of products addressed to consumers by as much as 12% within 3 years [28].

In Poland, the only conducted pilot studies on the benefits of short food supply chains so far have shown that consumers pay particular attention to the following [29]:

- Higher product quality—85%;
- Better health and nutritional values of food—65%;
- Favorable price–quality ratio—35.8%;
- Confidence in product origin—33.3%;
- Direct contact with the producer—25%;
- Higher availability of niche products—22.5%;
- Positive impact on the local economy—18.3%;
- Maintaining ties in society—12.5%.

According to B. Tundys [30], consumers in short food supply chains are more conscious of their choices. In addition, they have a relatively broad knowledge of the essence and the need to implement the principles of sustainable development in practice, and are also aware of the transformations taking place in the local food production markets as well as the importance of eating healthy and high-quality products [31].

The available research shows that consumers approach local products positively, perceiving them as fresh, originating from a reliable source, and more organic. At the same time, they are aware that by purchasing products directly from the local farmer, they contribute to making their farm a stronger one. They simultaneously show a declarative willingness to pay more for such a product [32]. The willingness to incur higher food purchase costs is strongly correlated with consumers’ lifestyles and values [33,34]. It is also determined by the age of the respondents (the older they are, the higher the tendency to purchase local goods) [35,36], income (higher) [37,38], or level of education (higher) [39,40]. The commitment to following a healthy lifestyle, caring for biodiversity, and concern for global climate change plays a significant role in this respect [41]. Moreover, people raising small children are more interested in local products [37].

An interesting discussion about territorial conditions is going on. In many studies, it has been recognized that SFSCs function better on the outskirts of large cities, where a larger population, as well as a potentially higher level of income and population density, result in a higher absolute territorial demand than in the case of small towns [42,43]. However, there is abundant evidence that the opposite is true—it is the inhabitants of villages or smaller towns who show a greater demand for local products than the respondents from large cities. Their consumer attitudes result from the direct perception of the relationship between the development of direct sales and the condition of farms or the local economy [44].

Therefore, apart from the group of consumers who approach local food presenting a rational distance (local does not have to be better) [45], there is a clearly distinguishable sphere of the market being subject to behavioral factors [46,47]. This is manifested in a strong belief that local agriculture is a reliable and trustworthy partner, drawing on local tradition and ensuring security in turbulent times [1,39,48,49].

In conclusion, the above source literature review leads to the conclusion that the research on short food supply chains shows a significant asymmetry in favor of supply-side analyses, with many fever studies covering the demand potential. In addition, many of these analyses are based on theoretical digressions rather than substantive empirical analyses [21]. This trend is relatively well developed in countries such as France or Italy [50,51], where direct sales has been a popular channel dedicated to selling food for years. Poland belongs to the group of countries where such studies are, firstly, scarce, and secondly, focused on the macro-scale assessment of demand rather than the research focused on the local market. The presented study has a chance to fill the research gap defined in the above way, to expand the knowledge about the potential demand for food sales within SFSCs in the local market dimension. This knowledge may also be useful in other countries, providing new evidence on the importance of behavioral, cultural, and institutional factors in shaping the development of short food supply chains [8].

## 3. Materials and Methods

### 3.1. Setting

In accordance with the assumption of territorial, organizational, and social proximity of SFSCs, the preference surveys were adapted to the respective, i.e., local size of the market. Finally, the area of Kamienna Góra county was selected for the study, due to its specific features of location, socio-economic conditions, and innovative activity in the field of direct sales development.

**Figure 1:**
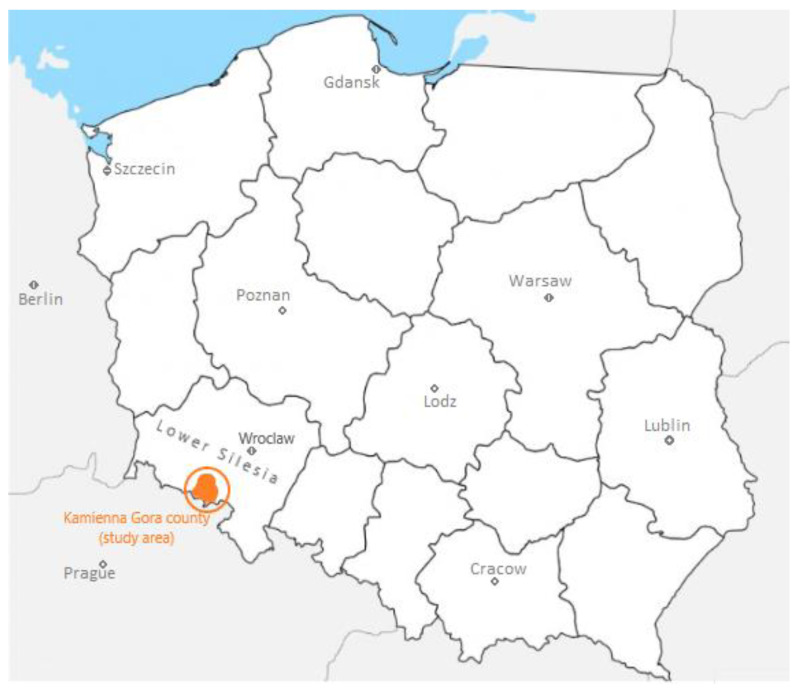
Location of the research area. Source: authors’ compilation.

The study area is located in the southern, mountainous part of the Lower Silesia region, situated peripherally at the border with the Czech Republic (Figure 1). Kamienna Góra is inhabited by 20,000 residents, while the entire county has a population of approximately 50,000 inhabitants, and due to its specific location in the mid-mountain valley, it is a good example of a closed spatial and hierarchical system. Therefore, the scope of the research coincides with the daily urban system [52], i.e., the natural system of gravity generated by the city of Kamienna Góra. In this space, the vast majority of daily migrations performed by the Kamienna Góra county residents take place, constituting one of the most important criteria for delimiting functional systems [53,54].

The structural problems of economic transformation related to the decline of the mining and light industry represent another feature of this area. As a consequence, the residents’ income is relatively lower. The average salary here is only 78% of the value for the Lower Silesia region (81% in relation to the entire Poland). The agricultural sector, due to difficult soil and climate conditions, is based mainly on breeding and milk production. However, the inability to compete with the areas more suitable for agriculture motivates farmers to search for market niches and new technological solutions. To meet these trends, a business incubator was created in this area, addressed to farmers and food producers, initiated and institutionally supported by the municipality of Kamienna Góra. To the authors’ knowledge, this is the first such initiative in Poland.

### 3.2. Study Design

As mentioned in the introduction, the existing research in Poland has been primarily focused on the supply potential, and therefore, the authors of this study took into account the demand aspect of the problem. At the same time, not only were consumer preferences analyzed, but also the state of knowledge about the possibilities of making purchases within the local food market. The study was conducted in the autumn of 2021.

### 3.3. Statistical Methods

The CAWI (Computer-Assisted Web Interview) method constituted the basis for the process of collecting research material. This technique consists in conducting research using survey questionnaires provided electronically. Owing to the Internet survey mode, online surveys can be carried out involving large groups of respondents, while ensuring their anonymity and the possibility of performing many parallel independent measurements, significantly reducing the time and cost of conducting the survey.

The draft survey form was subjected to external validation (ex ante). It consisted of its testing by 10 selected respondents. The opinions collected allowed clarifying the questions, eliminating terms that were ambiguous and incomprehensible to the average audience.

The dataset obtained in the survey process was subjected to basic methods of descriptive statistics. The use of more complex methods was considered inexpedient and would reduce the readability of the results. Graphical presentation of the results was presented using bar charts. This simple method allows clear visualization of the distribution of responses and preferences of respondents.

### 3.4. Data Sources

There is no validated instrument to measure the demand potential of the SFSC, so the authors created a survey referring to the prototype of the local sales platform. In this study, the authors have used the Google survey editing form, in which 30 substantive questions were formulated along with the particulars specifying: gender, number of people in the household, average monthly income per person in the household, age, education, and place of residence. In addition to questions about the idea of SFSC, part of the form (17 questions) focused on a prototype sales platform containing data about local producers and their offerings. The questions were of a mixed nature—some of them were provided in the form of a single-choice test, some allowed multiple choice answers, some were open-ended, and finally, some were based on a Likert scale (survey form attached below). Since the CAWI method ensures completing the questionnaires individually by the respondents, special attention was paid to the nature, conciseness, and clarity of the questions asked. A kind of innovation was the use of the LIBRUS application in this study, adopted, e.g., by schools for remote communication with students’ parents. The assumption was made, following the research conducted by T. Bech-Larsen and T.T. Jensen [55], that parents are the best target group for high-quality, traditional, and regional products. As it turned out, it was the most effective channel for reaching the respondents. Despite placing links to the survey also in local social media, the respondents were mainly parents of children from Kamienna Góra county.

### 3.5. Study Size

Taking into account the total number of children in schools, the answers were submitted by parents of 22.2% of them, which should be considered a representative result for this group of respondents. The answers were provided mainly by women (83%), living in multi-person households (1-person—3%, 2-persons—11%, 3-person—21%, 4-person—45%, ≥5-person households—19%). In terms of earnings, people with income in the range of PLN 1000–3000 per person (48% of respondents), aged 30–50 (72% of respondents), and with university education (60% of respondents) were the dominating ones. In light of the above presented research review, it can be presumed that this group of respondents is potentially most aware of the advantages of the local food market.

## 4. Results

The research has shown that the supply side in the study area is represented by approximately 600 farms, 20% of whom conduct direct sales within the framework of SFSCs, while another 50% do not perform such activity despite having the appropriate potential to do so [21]. Three direct sales initiatives are operating in the county, associating 23 agricultural producers and food processors.

**Figure 2:**
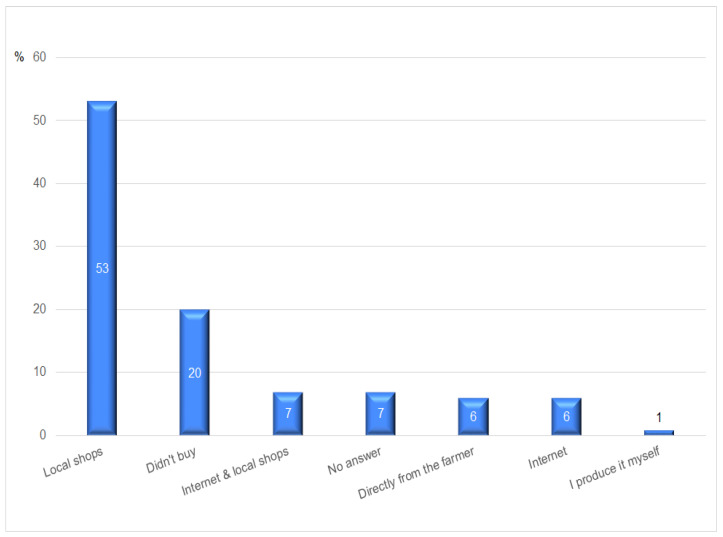
Purchases of local agricultural and food products. Source: authors’ compilation based on the collected data.

The first part of provided answers presents a rather ambiguous picture of the surveyed demand group’s commitment to local food markets. It turned out that 38% of the respondents are familiar with the idea of short food supply chains; however, only 22% have heard about online stores where local agricultural and food products from the Kamienna Góra county are sold. Only 20% of the respondents declared that they never buy local agricultural and food products. The remaining respondents indicated that they most often purchase local food products in local shops (53%), via the Internet and local shops (7%), or directly from the farmer (6%). It is clear that purchasing food directly from the farmer in this area occurs to a very limited extent. The results of all preferences are shown in Figure 2.

**Figure 3:**
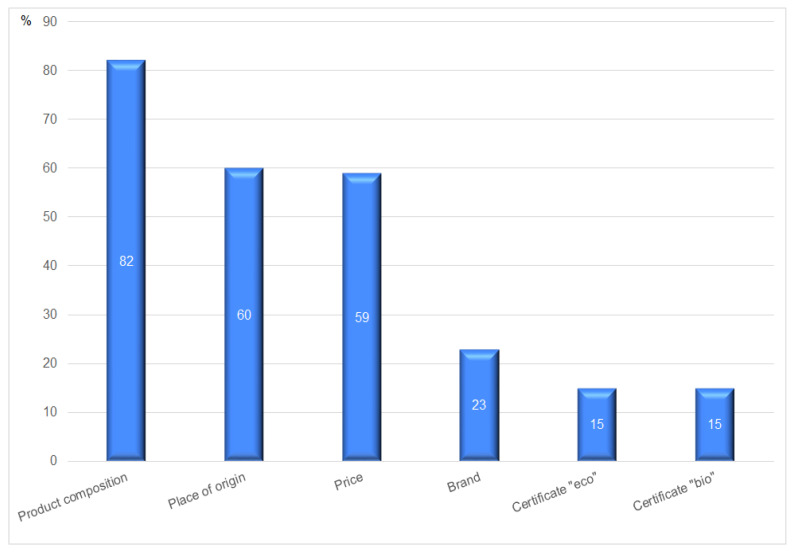
The main determinants decisive about the purchase of agri-food products. Source: authors’ compilation based on the collected data.

Figure 3 shows that product composition (82%) and its place of origin (60%) remain the essential determinants in purchasing agri-food products. Interestingly, the price of goods takes only the third position among shopping preferences. It reveals that local producers should emphasize the natural ingredients and production methods as well as guarantee the local origin of agricultural products. However, when assessing these characteristics, they rely more on the knowledge of producers and food processors than on the certificates they have obtained.

The research has also shown that the respondents most often shop for groceries two (38%) or three times per week (34%). This is confirmed by the fact that Poles, as compared to other Europeans, stand out regarding the frequency of shopping and a strong attachment to small shops close to their home. According to Nielsen’s reports [56], an average Pole visits small-format stores 12 times per month and also makes 13 purchases in supermarkets and several times in hypermarkets. Despite these habits, in this research, they declare their willingness to buy groceries in advance (50%—probably yes and 20%—definitely yes), which is crucial in the case of products traded within short food supply chains. It seems, therefore, that the respondents appreciate the value of local products, for which they would even be willing to change their shopping habits.

**Figure 4:**
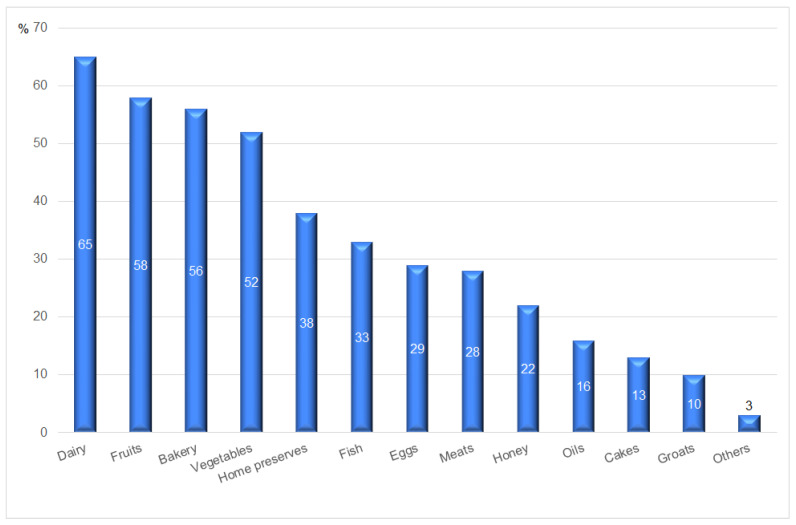
Consumer preferences for purchasing products in short food supply chains. Source: authors’ compilation based on the collected data.

When it comes to groups of local products, the respondents were most interested in buying dairy (65%), bread (56%), vegetables (52%), and fruit (58%). Detailed data are presented in Figure 4. This preference also seems to favor the development of local food markets which highlight the freshness of products resulting from a short supply chain. As a result, local goods do not need to be additionally preserved to extend their shelf life.

**Figure 5:**
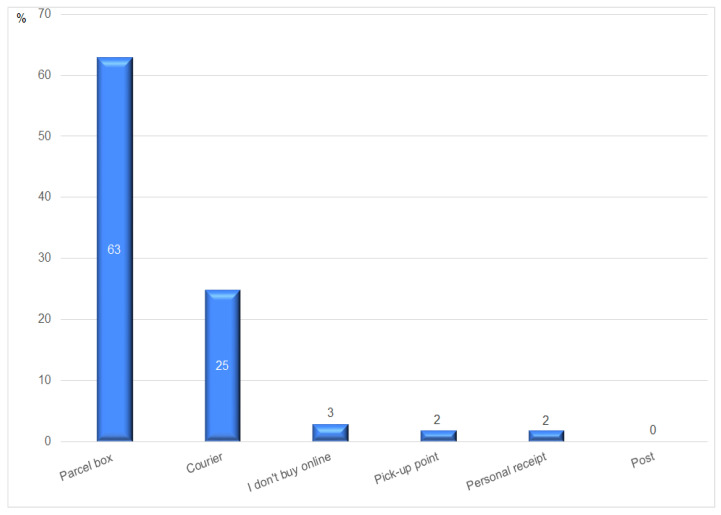
Deliveries of the agricultural and food products purchased via the Internet. Source: authors’ compilation based on the collected data.

Despite the small share of online food sales, the dynamics of this form of shopping are very high. Therefore, the authors asked what types of delivery are used by customers when shopping online. In this case, the Polish peculiarity on a global scale was confirmed. According to the Gemius study [57], for the first time in history, parcel lockers were more popular than couriers in terms of the delivery form most often chosen by Internet users when shopping online. In the presented study, the respondents indicated that when shopping online, they most often choose parcel lockers (63%). Deliveries by courier (25%) were ranked only second. It is worth noting, however, the distance in these preferences that separates these two forms of delivery. Only 2% of the respondents declare the fact of collecting products personally from the manufacturer. These results are illustrated in Figure 5.

In turn, the idea of an online store where local agri-food products are sold collectively is most often associated with a positive reception by respondents (75%), as is the mechanism of operation of the sales platform from zziemi.pl (72%). For 59% of respondents, the offer of products on the platform is satisfying, while prices are acceptable. For 74% of respondents, the available forms of payment and delivery on the platform are satisfactory, while for 71%, the visual side of the zziemi.pl website is attractive. In total, 75% of respondents appreciated the functionality of the website, while 81% appreciated its transparency.

**Figure 6:**
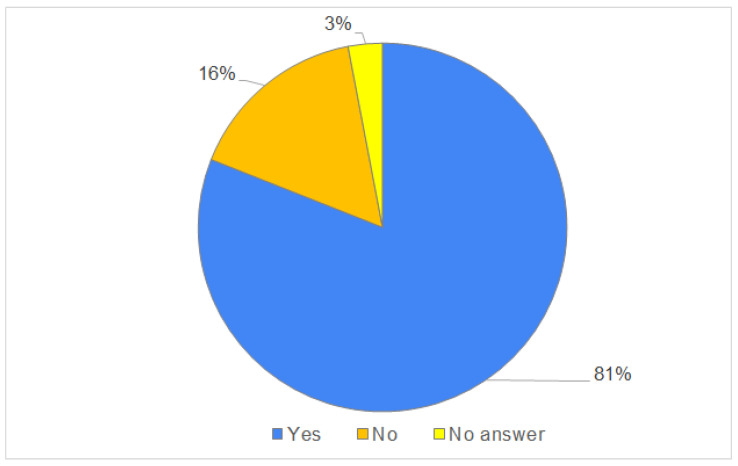
Willingness to purchase in the online marketplace. Source: [58].

The research showed that, after learning about the zziemi.pl platform’s offerings, as many as 81% of respondents said they were willing to shop on the platform (see Figure 6), especially since as many as 35% of respondents admitted that the pandemic had caused them to buy local products more often.

The results of the research showed that the awareness related to the functioning of local food markets is relatively high in the analyzed area, and only every fifth person has not used this method of buying agricultural products and food preserves so far. More than half of them are convinced that small local shops provide such an assortment. The idea of short food chains is also well identified—the result at the level of almost 40% in a specific area (not being a part of a large agglomeration) should be considered highly satisfactory. It seems, therefore, that the principle of shortening supply chains in the food market, promoted by the Ministry of Agriculture in Poland, brings about the right effect in this case. Perhaps this good result was influenced by the fact that the nationwide media campaign “Know and eat well” was carried out partly in this area. It showed many producers from both the Kamienna Góra county, as well as the initiatives of local governments and the business incubator of Kamienna Góra municipality, supporting the development of SFSCs in this region [59]. It seems that, on the one hand, this is proof of the effectiveness of such activities, and on the other, of the considerable demand potential presented by the local food market, even in the areas of lower profitability, located beyond the reach of large cities.

## 5. Discussion

This study is one of the few empirical analyses in Poland addressing this problem and remains in line with the results obtained in the, so far, pioneering work by Kawecka and Gębarowski [29].

The research shows that the surveyed community prefers home deliveries or a parcel locker, rather than purchasing directly from a farmer. The results, thus, confirm Poles’ inclination to buy local food products online using modern shopping tools, as well as their preference for delivery methods. Nationwide studies by Gemius [57] have shown that despite a small share of online food sales, the dynamics of this form of shopping are very high. Moreover, a very dynamic development of the parcel locker network, through which more than twice as many parcels are distributed than by traditional couriers is a typically Polish specificity. The development of this form of delivery remains in contrast with a very low propensity to collect goods in person—directly from the manufacturer.

The choice of the research target group—parents of children—is related to the opinion of Bech-Larsen and Jensen [55], who consider them potentially most aware of the advantages of the local food market. This research confirmed the opinion that people raising small children are more interested in local products [37]. The respondents, as in the studies by Adalja et al. [32], Renting et al. [33], and Hempel and Hamm [34], approach local products, perceiving them as fresh, from a reliable source, more organic, and by purchasing them directly from a farmer, they contribute towards strengthening their local economy. At the same time, they show a declarative propensity to pay a higher price for such a product.

It is worth noting that these findings may contribute to the global knowledge on this subject matter, providing new evidence confirming the importance of behavioral, cultural, or institutional factors in shaping the development of short food supply chains. As in the studies by Wallnoefer and Riefler [48], Berg and Preston [49], Campbell et al. [36], Feldmann and Hamm [39], and Bazzani et al. [1], the presented results confirm the respondents’ belief that local agriculture is a reliable and trustworthy partner, drawing on local tradition and guaranteeing security in turbulent times. In addition, the role of behavioral factors looks similar to the one presented in the studies by Migliore et al. [46] and Giampietri et al. [47].

Behavioral factors are also expressed in the very clear specificity of Poland as a country characterized by the highest frequency of shopping in Europe and a strong tendency to do shopping in small, local shops. This research confirmed that the respondents most often shop for groceries two or even three times a week. The high popularity of parcel lockers as a delivery form of goods purchased via the Internet is also a peculiar phenomenon on a global scale. The respondents surveyed in this study confirmed the Gemius findings [57] that parcel lockers are much more popular than courier services. Such factors have to be considered in comparative studies analyzing the situation in different countries. The authors believe that the presented research sheds new light on the greater propensity to purchase local goods by a higher-income group. Relatively high interest in SFSCs has been recorded by the authors in the area featuring lower average earnings than in the Lower Silesia region and throughout Poland. This contradicts the views expressed by Betz and Farmer [37] and Brown [38].

A similar situation occurs in terms of territorial conditions. Many authors recognize that SFSCs function better in the outskirts of large cities, where a bigger population as well as potentially higher income levels and population density result in higher absolute territorial demand than in the case of small locations [42,43,44]. The presented results contradict this belief, because the research area chosen by the authors is outside the range of larger cities, and yet the respondents showed high awareness of the importance of SFSCs and reported high demand for local products. In this respect, the authors of this research are rather similar in their opinions to those presented in the study by Kiss et al. [44].

In the authors’ opinion, this emphasizes the role of activities promoting such forms of purchase and sales. Both the choice of the research area and the relatively high awareness of the respondents result from the activities carried out by the local government. It took over the role of market organizer, focusing primarily on generating demand, based on the soft activities (i.e., promoting, advising, integrating, supporting, etc.) and the hard ones (entrepreneurship incubator, co-funding of the Internet platform, etc.). Therefore, it can be considered one of the best examples of the SFSCs model in Poland with the institutional participation of the local government.

## 6. Conclusions

The growing importance of the food supply chain concept, both in the world of theory and practice, is confirmed by the intensively developing research, which resulted in the emergence of several new problems of a conceptual and methodological nature. However, the studies conducted so far show a significant asymmetry in favor of the supply-side analyses, with much fewer analyses covering the demand potential. In addition, many of these studies are based on theoretical digressions rather than substantive empirical analyses. Poland belongs to the group of countries where very few studies of this type have been published. It can, therefore, be considered that the presented study fills this research gap, expanding the knowledge related to the potential demand for food sales in SFSCs in the dimension of the local market.

In conclusion, the presented research confirmed that both in Poland and in European countries such as France or Italy, there is a high demand potential for local agri-food products, and taking advantage of it depends on the active involvement of local governments in the creation of SFSCs. It seems that this observation can be considered one of the significant conclusions of this research. The authors have noticed that the undertaken topic has not yet found any suitable place in the source literature. A kind of return to a locality in terms of food markets can be effectively supported by the local government.

The presented research findings should also be perceived in light of certain limitations. Firstly, they covered a relatively undifferentiated research group (parents of school children). Secondly, they referred to the area of one county presenting high activity in the development of the local food market (although such a scale seems to be appropriate for analyzing markets where locality is the main feature). Both of these limitations can be considered as prospects for scaling the authors’ future research.

## Data Availability

The data presented in this study are available on request from the corresponding author.
